# The Book World of Medicine and Science

**Published:** 1894-03-24

**Authors:** 


### THE BOOK WORLD OF MEDICINE AND SCIENCE.

### BOOK REVIEWS.

THE CHILD, PHYSICALLY AND MENTALLY. By BERTHA MEYER. Translated by Friederika Salomon. (London: L. N. Fowler and Co.)

This book is a sort of "child's guide to knowledge;" it is intended, however, for the mother and not for the child; from an intellectual point of view it is perhaps better adapted to the mental capacities of the latter than the former. The writer starts with the assumption that her readers, presumably women, are fools, and she keeps up this supposition to the end of the work. At the commencement of the book she conducts her readers through the whole realm of science, as applicable to the laws of health, pausing to notice such diverse phenomena as combustion in our bodies, chemical forces in nature, human poison, the voices within us, the part minerals play, moisture in the air, snowstorm in a concert hall, and the child's first cry. We simply quote at random the descriptive headings of a few consecutive pages. She then passes on to review the question of nursing, feeding, doctoring, and the recreation of children. With some of her dictums we agree, others are calculated to do harm. The question of artificial versus natural feeding, Bertha Meyer, you have not properly studied—before you bring out another edition you must look up this question; you will find that scientific accuracy as to amount, quality, and uniformity, can only be attained by artificial methods. You are now living at the end of the nineteenth century, and you must remember that art is more accurate and reliable than nature. Moreover, your teaching on dietetics generally is not sound or up to date. Your method of feeding the puerperal woman is old-fashioned and very inadequate, and it is a pity to recommend an infant of ten weeks to commence farinaceous foods. Surely, on your own showing this is not nature; it certainly is not science. We like your remarks on physical education and kindergarten games, you have evidently had practical experience in these respects, and hence are in a better position to teach these subjects than many of the matters with which you deal.

PROTOPLASM AND MICROSCOPIC FORMS. By O. BÜTSCHLI, Professor of Zoology in the University of Heidelberg. Translated by E. A. MINCHIN, B.A., Fellow of Merton College, Oxford. (Adam and Charles Black.)

One of the most important problems which await solution at the hands of biologists, zoologists and botanists alike, is that which is concerned with the physical structure of protoplasm. We possess a certain amount of knowledge respecting the chemical composition of this elusive body; we are acquainted with the elements which occur in it, and we can even go so far as to enumerate with some confidence many of the highly complex substances which are invariably associated with it. What at present we cannot do, at least otherwise than tentatively, is to frame a physical conception of the manner in which the chemical compounds are grouped, to determine the *physical conformation* of the constituents on which, in part at any rate, those characteristics vaguely referred to as vital depends.

Almost every writer on protoplasm—and there have been many—has evolved his own hypothesis as to its nature; but these views have for the most part failed either to fit in with the observed structure, or to provide a consistent interpretation of the phenomena.

Bütschli, however, has framed a theory which forms a considerable advance on its predecessors, and this is true quite irrespective of the verdict which will ultimately be passed by posterity on it. The great merit of his work lies, not only in the brilliant conclusions which he draws, but also in the way by which he arrives at them. Leaving the field of loose speculation, he has investigated the structure of protoplasm in the light of substances whose physical properties are understood, and which can form the subject of critical and decisive experiment. He has investigated the unknown in the light of the known, and his labours have been well rewarded.

Most of the more modern writers distinguish in protoplasm a fibrillar meshwork, which is bathed in a more fluid matrix. Bütschli, while admitting the microscopic evidence on which the fibrillar theory is based, does not believe that it interprets correctly the facts as observed.

He gives in his book an excellent and critical account of the chief divergent views which have been put forward by the most prominent investigators, and the general result of the discussion is to bring out clearly that many of the important facts connected with living cells cannot be explained on the assumption of a fibrillar network in the protoplasm. He himself advocates the view that the true explanation of the microscopic image lies in the conception of protoplasm, as consisting of a fine froth or foam.

Such foams may be manufactured by the interaction of two mutually insoluble liquids under certain conditions. For example, if thick olive oil be properly rubbed up with potassium carbonate, and a drop of the mixture be placed in water, a foam is produced which imitates the characters of protoplasm in a most remarkable way. The drop now appears turbid, and examined under the microscope, exhibits an appearance of a reticulum, which careful reflexion shows must really be the optical expression of a honeycomb or alveolar structure. The walls of the "honeycomb" are formed by thin lamellæ of oil, and the cavities are filled by the watery solution. Now so striking is the resemblance which exists between such a foam and genuine protoplasm that it is possible to deceive even a practised observer as to its real nature; and the similarity goes still further; just as movement takes place in living protoplasm so also does it occur in the oil foam, which, in successful preparations, may easily be mistaken for an amœba.

Bütschli then explains the structure of protoplasm as possessing essentially the same physical properties as a microscopic fluid foam; movements, of translation and streaming, are observable in both, and are in both cases due to changes of surface tensions. Pseudopodia are produced in this way by the bursting of superficial alveoli, for the escape of their contents alters the surface tension in the vicinity of the point of rupture. It is, of course, not difficult to imagine further that chemical action going on in such a highly complex body as protoplasm, might constantly alter the surface tensions at various points, and that these changes would then be followed by movements.

It is perhaps natural that many persons should regard with disapproval, or even dislike, an attempt to explain the complex nature of protoplasmic structure and movement by a recourse to the phenomena exhibited by mere oil foams. Undoubtedly the chemical nature of the two bodies are widely dissimilar, but from a physical point of view it cannot be questioned that the comparison is a helpful one, and that Professor Bütschli has rendered good service in enabling us to form a conception of protoplasm, which renders much that has hitherto been obscure, even if it leaves other difficulties still unexplained.

The original work is too well known, even though it has been but recently published, to need further comment, but its appearance in English will be welcome to many whose knowledge of German is hardly sufficient to enable them to grapple with a somewhat technical subject. It seems, then, perhaps a little ungracious to pick out faults in the translation, especially when Mr. Minchin has performed a difficult task so well; but should a second English edition be called for, we should prefer to see the word *Hautschicht*, as applied to a vegetable cell, rendered by "ectoplasm" and not "cuticular layer," a term already appropriated to another structure. Again, the use of chemical symbols in the text when used merely to designate substances is not pleasing to an English reader. But these are small faults, and the English public should be grateful for easy access to the most remarkable book on the subject of protoplasm which has appeared for many years.

